# *In vitro* and *in vivo* antidiabetic potential of extracts and a furostanol saponin from *Balanites aegyptiaca*

**DOI:** 10.1080/13880209.2017.1343358

**Published:** 2017-06-28

**Authors:** Shahira Mohammed Ezzat, Amira Abdel Motaal, Sally Abdel Wanees El Awdan

**Affiliations:** a Department of Pharmacognosy, Faculty of Pharmacy, Cairo University, Cairo, Egypt;; b Department of Pharmacology, National Research Centre, Giza, Egypt

**Keywords:** Diabetic complications, pregnanes, insulin, C-peptides, desert date

## Abstract

**Context:***Balanites aegyptiaca* Del. (Zygophyllaceae) fruits are well-known antidiabetic drug in Egyptian folk medicine. Nevertheless, its mechanism of action is still unclear.

**Objectives:** Searching for the possible mechanisms of action of the plant and identification of its bioactive compounds.

**Materials and methods:** A bio-guided protocol based on the evaluation of α‐glucosidase (AG) and aldose reductase (AR) inhibitory activities was adopted to isolate the biologically active compounds from the methanol extract (MeEx). An *in vivo* antidiabetic study was conducted for the active extract, fraction and compound using streptozotocin-induced diabetic male albino Wistar rats at two dose levels (100 and 200 mg/kg.b/wt) for 2 weeks.

**Results:** Three compounds were isolated and identified: a sterol, (**1**) stigmasterol-3-*O*-β-d-glucopyranoside; a pregnane glucoside, (**2**) pregn-5-ene-3β,16β,20(R)-trio1-3-*O*-β-d-glucopyranoside; a furostanol saponin, (**3**) 26-(*O*-β-d-glucopyranosyl)-22-*O*-methylfurost-5-ene-3β,26-diol-3-*O*-β-d-glucopyranosyl-(1 → 4)-[α-l-rhamnopyranosyl-(1 → 2)]-β-d-glucopyranoside. Only compound **3** possessed significant AG and AR inhibitory activities (IC_50_ = 3.12 ± 0.17 and 1.04 ± 0.02 μg/mL, respectively), while compounds **1** and **2** were inactive. The *in vivo* antidiabetic study revealed that MeEx and furostanol saponin **3** possessed significant activities at a dose of 200 mg/kg through reducing the fasting plasma glucose level by 46.14% and 51.39%, respectively, as well as reducing the total cholesterol by 24.44% and 31.90%, respectively. Compound **3** also caused increment in insulin and C-peptide levels by 63.56% and 65%, respectively.

**Discussion and conclusions:** We presented a scientific base for using *Balanites aegyptiaca*, and shed the light on one of its saponins, as an antidiabetic agent in fasting and postprandial hyperglycaemia along with the improvement of diabetic complications.

## Introduction

Diabetes mellitus (DM) is a chronic disease which is usually associated with cases of morbidity, mortality and high health care costs (Sheetz and King 2002). According to the reports of the International Diabetes Federation (IDF), 415 million people have diabetes worldwide among which more than 35.4 million people are in the Middle East and North Africa. This number is expected to rise to 72.1 million by 2040. In 2015, more than 7.8 million cases of diabetes were reported in Egypt. Diabetic patients experience various vascular complications, such as atherosclerosis, diabetic nephropathy and neuropathy. Moreover, hyperglycaemia and hyperlipidemia are two important characters of diabetes (Sheetz and King 2002). Another complication secondary to hyperglycaemia is the poor metabolism of sorbitol which causes its accumulation in the tissues, such as the retina of the eye leading to loss of clarity in the lens. Aldose reductase (E.C.1.1.1.21) is an enzyme of the aldo-keto reductase family which catalyzes the reduction of glucose into sorbitol utilizing the cofactor NADPH (Yabe-Nishimura 1998). Therefore, inhibition of aldose reductase (AR) provides a potential therapeutic approach towards diabetes associated complications.

A therapeutic approach for treating diabetes is to control the postprandial hyperglycaemia by retarding the absorption of glucose. α-Glucosidase (EC 3.2.1.20) is located in the brush-border surface membrane of intestinal cells. The inhibition of its catalytic activity led to the retardation of glucose absorption and the decrease in postprandial blood glucose level (Robinson et al. 1991). Thus, α-glucosidase (AG) inhibitors reversibly delay the absorption of sugar from the gut (Campbell et al. 1996).

*Balanites aegyptiaca* (L.) Del. (Zygophyllaceae), commonly known as the desert date, is a spiny evergreen tree which grows in the arid regions of Africa, the Middle East, and southern Asia (Chothani & Vaghasiya 2011). In Egyptian folk medicine, the fruits are used as an oral hypoglycaemic drug (Kamel 1998). A previous *in vitro* study, carried out by a member of our group, proved that different extracts of *Balanites* fruit (made by extracting with methanol, cold water, hot water, and 70% ethanol) exhibited comparable glucose uptake effect to that of insulin in peripheral C_2_C_12_ skeletal muscle cells (Motaal et al. 2012). Another study proved the aldose reductase inhibitory activity of the saponin-rich fraction of *Balanites* fruits (Abdel Motaal et al. 2015).

In the present study, we designed a bio-guided fractionation protocol for the methanolic extract of *Balanites* fruits according to AG and AR inhibitory potential with the target of determining the capability of the plant in improving postprandial hyperglycaemia and diabetic complications. Moreover, isolation of the biologically active compounds responsible for the activity of the plant was carried out. A confirmatory *in vivo* study was also conducted for the active extract, fraction and one compound in streptozotocin-induced diabetic rats to evaluate their antihyperglycaemic and antihyperlipidemic activities, and their effect on insulin secretion.

## Materials and methods

### Chemicals and instruments

α-Glucosidase enzyme from Brewer's yeast (EC 3.2.1.20), the substrate *p*-nitrophenyl α-d-glucopyranoside (p-NPG), Na_2_HPO_4_·7H2O, NaH_2_PO_4_ (phosphate buffer, PH 6.8), β-NADPH, DMSO, streptozotocin and quercetin (positive control) were purchased from Sigma-Aldrich (St, Louis, MO). The positive control, acarbose, was purchased from Bayer Pharmaceuticals Pty, Ltd. (Wayne, NJ). Human recombinant aldose reductase and dl-glyceraldehyde were purchased from Wako Pure Chemical Industries, Ltd. (Osaka, Japan). Gliclazide was obtained from Servier Canada (Laval, QC, Canada). Pre-coated silica plates 60 GF_254_ (20 × 20 cm) (Fluka, Steinheim, Germany) were used for thin-layer chromatography (TLC). Silica gel H (Merck, Darmstadt, Germany) and Lichroprep RP-18 silica gel (15–25 μm, Merck, Darmstadt, Germany) were used for vacuum liquid chromatography (VLC). Silica gel 60 (70–230 mesh ASTM, Fluka, Steinheim, Germany), Diaion HP 20 (Sigma-Aldrich, St. Louis, MO), and Sephadex LH-20 (Sigma-Aldrich, Steinheim, Sweden) were used for column chromatography (CC). Spots were visualized by spraying with *p*-anisaldehyde/sulphuric acid. HR-FABMS was measured in the JOEL JMX-AX 505 (Joel Inc., Tokyo, Japan), HAD mass spectrophotometer at an ionization voltage of 70 eV. ^1^H NMR and ^13^C NMR spectra were recorded on a Bruker high-performance digital FT-NMR spectrophotometer operating at 400 (^1^H) and 100 (^13 ^C) MHz in DMSO-*d*_6_ as a solvent and chemical shifts were given in *δ* (ppm) relative to solvent as an internal standard.

### Plant material

The fruits of *B. aegyptiaca* were brought from Aswan in the summer of 2012. The plant was authenticated by Dr. M. Gebali, Plant Taxonomy and Egyptian Flora Department, National Research Center, Giza, Egypt. A voucher specimen (voucher no. 201) was deposited at the herbarium of the Pharmacognosy Department, Faculty of Pharmacy, Cairo University, Cairo, Egypt.

### Extraction and fractionation

The powdered pericarps of *B. aegyptiaca* (1 kg) were macerated in methanol on cold (2 × 4 L) until exhaustion. The extract was evaporated under reduced pressure at 40 °C to yield 650 g of dried methanol extract (MeEx). MeEx (600 g) was suspended in distilled water and subjected to liquid–liquid fractionation using chloroform and *n*-butanol saturated with water. Fractions were separately concentrated under reduced pressure to yield 16.14, 150.68 and 400 g of the chloroform (ClFr), *n*-butanol (BuFr) and residual water (AqFr) fractions, respectively. BuFr (100 g) was further fractionated over Diaion HP 20 column using increasing percentages of methanol in water. The fractions were separately concentrated under reduced pressure to yield D1, D2, D3, D4, D5 and D6 weighing 2, 5.8, 14, 20.6, 9.1 and 3 g, respectively.

### *In vitro* enzyme inhibition assays

AG and AR inhibitory activities of MeEx, ClFr, BuFr, AqFr, as well as the Diaion fractions D1–D6 were tested. AG inhibition was measured spectrophotometrically in a 96-well microplate reader using a procedure reported by Li et al. (2009). Acarbose was used as a positive control. The AR (EC 1.1.1.21) inhibitory activity was measured according to the procedure previously reported (Nishimura et al. 1991). The experiments were repeated three times, and the IC_50_ values were calculated and expressed as mean ± standard error.

### Isolation and structural elucidation of compounds 1–3

**Figure 1.**
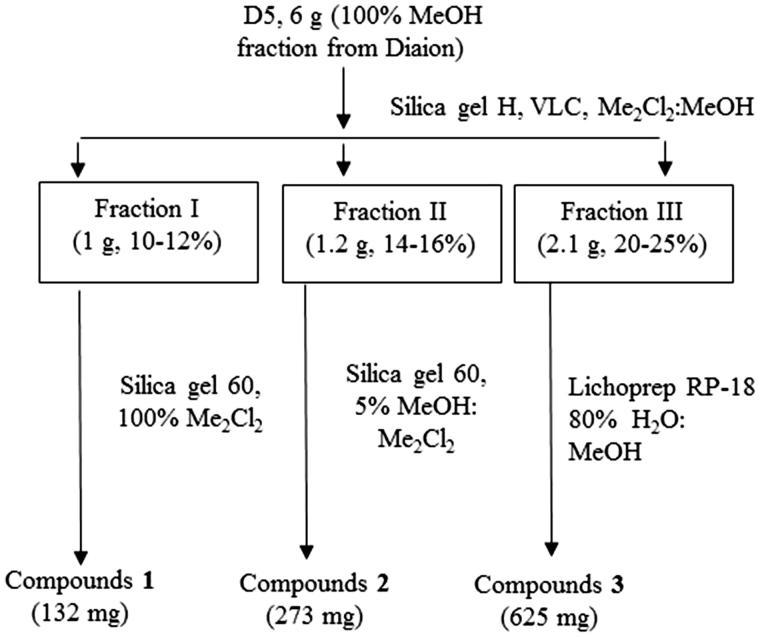
Scheme demonstrating the fractionation of the most bioactive fraction (D5) of *Balanites aegyptiaca*.

The most bioactive fraction D5 was fractionated and three compounds (**1**–**3**) were isolated (Figure 1). Spectral data of compounds (**1**–**3**) are found in the supplementary material, and Tables 1S and 2S.

**Table 1.** Inhibitory effects of the extracts, fractions and isolated compounds from *Balanites aegyptiaca* on *α*-glucosidase (AG) and aldose reductase (AR) enzymes.

	IC_50_ (μg/mL)	
Tested sample	AG inhibition	AR inhibition
MeEx	54.25 ± 0.17	21.63 ± 0.73
ClFr	406.30 ± 0.92	40.30 ± 0.92
BuFr	23.14 ± 1.08	13.64 ± 3.12
AqFr	37.67 ± 0.12	14.92 ± 2.01
D1	–	30.00 ± 4.61
D2	–	18.36 ± 0.82
D3	–	13.87 ± 1.42
D4	–	10.65 ± 0.75
D5	05.19 ± 0.49	05.60 ± 0.49
D6	–	23.00 ± 3.52
Compound **1**	407.70 ± 0.36	28.55 ± 1.06
Compound **2**	–	12.22 ± 0.71
Compound **3**	03.12 ± 0.17	01.04 ± 0.02
Acarbose	24.00 ± 2.31	–
Quercetin	–	03.00 ± 4.25

Results are expressed as mean ± standard error, *n* = 8.

### *In vivo* antidiabetic activity

Male albino rats (Wistar strain, 150–200 g) and male mature albino mice of 20–25 g were obtained from the animal house colony of the National Research Center, Egypt. The experimental protocol followed the Institutional Animal Ethical Committee of the National Research Centre. The acute toxicity studies were performed on the mice. They were given different doses of MeEx, BuFr and compound **3** up to 2000 mg/kg and were observed for 24 h for mortality. The animals survived and then four additional animals were tested sequentially so that a total of five animals were tested.

DM was induced by a single i.p. injection of STZ (55 mg/kg) in rats (Brosky and Logothetopoulos 1969). Only rats with serum glucose levels of more than 250 mg/dL were selected and considered diabetic animals. MeEx, its active fraction (BuFr) and the active compound **3** were tested in the STZ-induced diabetic rats at two dose levels (100 and 200 mg/kg.b/wt) for 2 weeks. At the end of the 2-weeks treatment period, the animals were kept for an overnight fasting and the blood samples were collected from the retroorbital plexus and allowed to clot for 30 min at room temperature. These blood samples were centrifuged at 5000 rpm for 20 min and the serum was separated and stored at −80 °C until analysis was done. Serum samples were analyzed spectrophotometrically for serum glucose, triglycerides and cholesterol. Serum glucose was estimated by glucose oxidase–peroxidase method test agent kit (Stanbio, Boerne, TX) (Cheyne and Gilmore 1973). Triglycerides were estimated by enzymatic methods by using a diagnostic kit (Biodiagnostic, Cairo, Egypt) (Fossati and Prencipe 1982). Total cholesterol was estimated by enzymatic methods by using a diagnostic kit (Biodiagnostic, Cairo, Egypt) (Allain et al. 1974). Serum insulin was estimated by a radioimmunoassay technique using the ALPCO Insulin (Rat) ELISA kit (Judzewitsch et al. 1982). Serum C-peptide was measured with a rat insulin enzyme-linked immune absorbent assay kit (C-Peptide EIA Kit, Sigma-Aldrich, St. Louis, MO). Results were expressed as mean ± SEM for biochemical estimations. The quantitative data were analyzed by ANOVA followed by Tukey multiple comparison tests. The Kruskal–Wallis test was used for the quantitative analysis of the histopathology score values. Results were considered to be significant at *p*  < 0.05.

## Results

### *In vitro* enzyme inhibition assays

The MeEx of *Balanites* showed remarkable inhibition of AG and AR, so it was subjected to further fractionation where the BuFr showed the highest AG and AR inhibitory activities (with the lowest IC_50_ values; 23.14 ± 1.08 and 13.64 ± 3.12 μg/mL, respectively) (Table 1). The BuFr was further fractionated over Diaion HP column to give six subfractions, among which only D5 possessed an AG inhibitory activity (IC_50_ =  5.19 ± 0.49 μg/mL) (Table 1). All the Diaion fractions (D1–D6) exerted AR inhibitory activities; however, D5 was the most active (IC_50_ value = 5.60 ± 0.49 μg/mL compared with 3.0 ± 4.25 μg/mL of the reference standard quercetin) (Table 1).

### Structural elucidation and bioactivity of compounds 1–3

**Figure 2.**
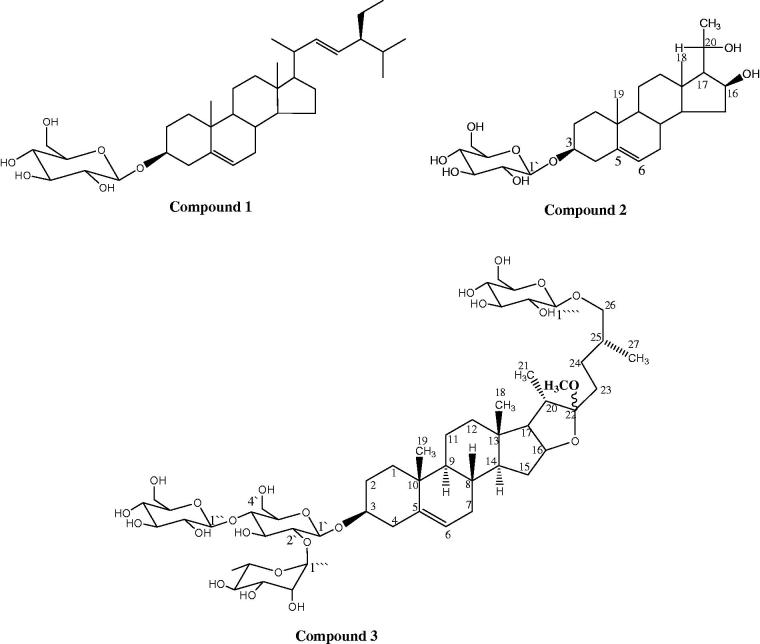
Structures of the isolated compounds.

Purification of D5 yielded three compounds (**1–3**) (Figure 1). Compound **1** was identified as stigmasterol-3-*O*-β-d-glucopyranoside (stigmast-5,22-dien-3-*O*-β-d-glucopyranoside) by comparing its spectral data (Supplementary material, Table 1S) with the previous reports (Ridhay et al. 2012). The high-resolution HRESI-MS of compound **2** in negative mode gave a molecular ion peak [M-H]^−^ at *m*/*z* 495, consistent with the molecular formula C_27_H_44_O_8_. The chemical shifts of the aglycone moiety could be superimposed on those reported for pregn-5-ene-3β,16β,20(*R*)*-*triol (Kamel and Koskinen 1995). The downfield shift of C-3 (*δ*_C_ 77.6 ppm) as well as the upfield shifts of C-2 and C-4 (*δ*_C_ 30.1 and 37.0 ppm, respectively) in the ^13^C NMR spectrum of compound **2** (Table 1S) indicated that it is a glycoside with one sugar moiety attached to C-3. The large coupling constant (*J* = 7.0 Hz) at *δ*_H_ 5.18 ppm for the anomeric proton in the ^1^H NMR spectrum indicated a β-configuration sugar. Therefore, compound **2** was identified as pregn-5-ene-3β,16β,20(*R*)-triol 3-*O*-β-d-glucopyranoside (Figure 2).

The HRESI-MS of compound **3** showed [M-H]^−^ at *m*/*z* 1077. ^1^H NMR and ^13^C NMR data of the compound **3** showed four signals for quaternary carbons corresponding to C-5, C-10, C-13 and C-22 of a furostan (Hosny et al. 1992). A singlet at *δ*_H_ 3.14 with a direct correlation with the peak at *δ*_C_ 47.5 ppm in HSQC indicated the presence of a methoxy group. The attachment of the methoxy group to C-22 (*δ*_C_ 113.7 ppm) was deduced from the HMBC correlations. The downfield shift of C-22 (*δ*_C_ 113.7 ppm) confirmed that it is a C-22 hydroxy furostane derivatives (Agrawal et al. 1985). Four doublets appeared at *δ*_H_ 5.24 *d, J* = 1.2 Hz (H1‴), 4.51, *d,J* = 7.6 Hz (H1′), 4.40, *d, J* = 7.6 Hz (H1″) and 4.24, *d, J* = 7.7 Hz (H1″″), assigned to four anomeric protons. These four signals showed a direct HSQC correlation with four carbon signals that appeared at *δ*_C_ 100.5, 104.5, 102.2 and 104.7 ppm for C1′, C1″, C1‴ and C1″″, respectively (Table 2S). A doublet at (1.05, *J* = 6.7 Hz) was attributable to the CH_3_-6 of rhamnose. Furthermore, ^13 ^C NMR data also showed the peaks of three glucose units and one rhamnose unit having its methyl group at *δ*_C_ 17.9 (C-6‴). The positions of the four sugar units were determined by the cross peaks in the HSQC, HMBC and COSY spectra. The glycosylation of C-3 could be detected from a correlation between C-3 (*δ*_C_ 79.2 ppm) and H-1′ in HMBC. Another attachment was deduced from HMBC correlation between C-2′ (*δ*_C_ 78.7 ppm) and H1‴, in addition to C-4′ (*δ*_C_ 81.0 ppm) and H-1″, which indicated that C4′ and C-1″ were attached through an oxygen linkage. Finally, C-26 (*δ*_C_ 76.0 ppm) showed HMBC correlation to H1″″, which indicated the attachment of C-1″″ to C-26 through an oxygen atom. The glucose units were assigned to have β configuration from their large coupling constants (*J* = 7.6–7.7 Hz). On the contrary, the rhamnose configuration was deduced to be α-from the small coupling constant (*J* = 1.2 Hz). The absolute configuration of the sugars was determined according to the method of Hara et al. (1987). This compound was identified as 26-(*O*-β-d-glucopyranosyl)-22-*O*-methylfurost-5-ene-3β,26-diol-3-*O*-β-d-glucopyranosyl-(1 → 4)-[α-l-rhamnopyranosyl-(1 → 2)]-β-d-glucopyranoside (Figure 2, Table 2S).

**Table 2.** Results of the *in vivo* study on STZ-induced diabetic rats.

Group	Glucose level (mg/dL)	Triglycerides (mg/dL)	Cholesterol level (mg/dL)	Insulin (μIU/mL)	C-peptide (ng/mL)
Normal	94.87 ± 7.39^b^	105.3 ± 9.17	115.9 ± 5.86	4.315 ± 0.355^b^	2.900 ± 0.160
Control (diabetic)	251.4 ± 18.07^a^	144.2 ± 13.46	168.0 ± 6.26^a^	1.15 ± 0.03^a^	0.715 ± 0.02^a^
MeEx (100 mg/kg)	154.4 ± 13.22^b^	124.1 ± 6.19	130.6 ± 1.27^b^	2.72 ± 0.17^a^^,^^b^	1.50 ± 0.05^a^
MeEx (200 mg/kg)	135.4 ± 11.33^b^	107.9 ± 7.28	126.1 ± 8.17^b^	3.56 ± 0.31^b^	1.90 ± 0.08
BuFr (100 mg/kg)	232.3 ± 20.18^a^	125.4 ± 11.08	139.2 ± 4.16	1.48 ± 0.04^a^	1.33 ± 0.08^a^
BuFr (200 mg/kg)	226.8 ± 20.61^a^	107.9 ± 7.28	120.5 ± 4.14^b^	1.68 ± 0.02^a^	1.46 ± 0.16^a^
Compound **3** (100 mg/kg)	207.4 ± 18.33^a^	112.2 ± 9.89	139.2 ± 10.30	1.99 ± 0.04^a^	1.42 ± 0.07^a^
Compound **3** (200 mg/kg)	122.2 ± 18.22^b^	113.5 ± 7.73	115.6 ± 7.12^b^	3.17 ± 0.28^b^	2.04 ± 0.06^b^
Gliclazide (10 mg/kg)	93.09 ± 5.80^b^	103.7 ± 9.73	125.3 ± 5.98^b^	2.82 ± 0.18^a^^,^^b^	1.78 ± 0.11

Each value represents mean ± SEM. *n* = 6. One‐way ANOVA followed by Tukey’s multiple comparison test.

a Represent statistical significance versus normal (*p* < 0.05).

b Represent statistical significance versus control (*p* < 0.05).

Testing the inhibitory activity of the *Balanites* isolated compounds (**1**–**3**) on AG enzyme (a disaccharidase) showed that only the furostanol saponin (compound **3**) had a significantly high activity (IC_50_ = 3.12 ± 0.17 μg/mL) compared with acarbose tested under the same conditions (IC_50_ = 24 ± 2.31 μg/mL) (Table 1). Similarly, only compounds **3** showed a significant AR inhibitory activity (IC_50_ =1.04 ± 0.02 μg/mL) compared with 3.0 ± 4.25 μg/mL of the reference standard quercetin (Table 1). Whereas other compounds isolated from *Balanites* active fraction, the stigmasterol glycoside (**1**) and the pregnane glycoside (**2**) possessed moderate activities (IC_50_ = 28.55 ± 1.06 and 12.22 ± 0.71 μg/mL, respectively).

### Acute toxicity study

The acute toxicities of the three samples (MeEx, BuFr and compound **3**) were studied where the mice took ascending oral doses up to 2000 mg/kg of each sample. They were observed closely for 24 h and daily for 14 days, where no mortality was observed. Hence 200 mg/kg (1/10 of the 2000 mg/kg) was selected as a maximum safety dose, in addition to a descending dose of 100 mg/kg body weight.

### *In vivo* antidiabetic activity

Compound **3**-treated group (200 mg/kg) showed 51.39% reduction in fasting blood glucose level of the rats. The MeEx reduced the fasting plasma glucose level by 38.58 and 46.14%, at the two dose levels 100 and 200 mg/kg, respectively.

MeEx significantly reduced the cholesterol level by 22.26 and 24.44% at 100 and 200 mg/kg, respectively. Compound **3** induced a 31.19% reduction in the cholesterol level at 200 mg/kg compared with Gliclazide which caused a 25.42% reduction (Table 2).

Compound **3** (200 mg/kg) also caused an increase in insulin and C-peptide levels by 63.56 and 65%, respectively. While the parent MeEx increased the insulin level by 67% at 200 mg/kg (Table 2).

## Discussion

The ultimate goal of diabetic patient treatment is to maintain near normal levels of glycemic control, in both the fasting and post-prandial states. Many natural resources were investigated with respect to suppression of glucose production from carbohydrates in the gut or glucose absorption from the intestine (Matsui et al. 2007).

Significant production of polyol in the lens during sugar-induced cataractogenesis stimulated great interest in the pathological role of the pathway in the development of cataract (Patel and Mishra 2009). Aldose reductase was found to be the primary factor responsible for this pathological condition (Patel and Mishra 2009). There were many reports of AR inhibiting activity of natural products such as *Mangifera indica* L. (Anacardiaceae)*, Eugenia borinquensis* Britton, *Eucalyptus deglupta* Bl. and *Syzygium malaccense* (L.) Merr. (Myrtaceae) (Guzmán and Guerrero 2005). The present study provided a new and powerful support for the use of *Balanites* and its furostanol saponin (**3**) in improving the postprandial hyperglycaemia through inhibition of AG and preventing some diabetic complications such as cataract through AR inhibition.

To confirm our results, an *in vivo* study was performed on the active samples (MeEx, BuFr and compound **3**). STZ-induced diabetes in rats is an accepted model for investigation of antidiabetic effects and mechanism of action of any novel antidiabetic agent (King 2012). Gliclazide was used as the standard drug in the present study. It has been proposed that sulphonylureas exert antihyperglycaemic effect through secretion of insulin from pancreatic β-cells and enhancement of insulin action on target tissues (Jackson and Bressler 1981).

In our study, there was a significant elevation in fasting blood glucose level in the STZ-diabetic group when compared with the normal group. MeEx and compound **3**-treated groups exhibited significant reduction of fasting plasma glucose level as compared with the diabetic group. The most commonly observed lipid abnormalities in diabetes are hypertriglyceridemia and hypercholesterolemia (Shirwaikar et al. 2005). The abnormal high concentration of serum lipid in diabetic animals is mainly due to an increased mobilization of free fatty acids from peripheral fat depots (Murugan and Pari 2006). In the present study, a significant increase in the total serum cholesterol in STZ-diabetic rats was noticed. This was due to the reduction in insulin levels which had a potent inhibitory effect on lipolysis, hence insulin deficiency was associated with an increased influx of fatty acids (Rajalingam et al. 1993). Administration of both doses of MeEx could significantly reduce cholesterol level, while the effect of the furostanol saponin (compound **3**) was more pronounced. Insulin and C-peptide are the products of enzymatic cleavage of proinsulin and are secreted into the circulation in equimolar concentrations. The estimation of serum insulin concentration alone may not be sufficient to confirm that the insulin has been secreted by the pancreas, because insulin undergoes significant first-pass clearance by the liver after being released from the pancreas. Therefore, estimation of both the concentrations of C-peptide and insulin was reported to be a valuable index of insulin secretion (Juárez-Rojop et al. 2012). The concentrations of serum insulin and C-peptide increased significantly in the groups treated with furostanol saponin (compound **3**). Meanwhile, MeEx had a positive effect on the insulin level but it showed a non-significant effect on the C-peptide level.

In conclusion, *B. aegyptiaca*, the famous Egyptian desert dates which have an ethnopharmacological reputation as an antidiabetic drug, could be used as a potential source of furostanol saponins for treating postprandial hyperglycaemia through its specific inhibitory effect on the α-glucosidase enzyme. *Balanites aegyptiaca* may also contribute to the improvement in the parameters associated with secondary complications of diabetes, through the inhibition of aldose reductase by its active furostanol saponin. The methanolic exract of *B. aegyptiaca* (MeEx) and the furostanol saponin (**3**) exerted *in vivo* antidiabetic effects through increasing the biosynthesis of insulin, and through their antihypercholesterolemic effects. These findings revealed scientific evidence for the therapeutic benefit of *B. aegyptiaca* in the management of diabetes and its acquired complications.

## Supplementary Material

Shahira_Mohammed_Ezzat_et_al_supplemental_content.zip
