# Supplementary material for: In vitro and in vivo antidiabetic potential of extracts and a furostanol saponin from Balanites aegyptiaca
Source: Pharm Biol. 2017 Jun 28;55(1):1931–6. doi: 10.1080/13880209.2017.1343358 (PMC6130680; doi:10.1080/13880209.2017.1343358)
Supplement: Shahira_Mohammed_Ezzat_et_al_supplemental_content.zip [file IPHB_A_1343358_SM9813.zip › Shahira Mohammed Ezzat et al supplemental content.docx]

**Supplementary Material**

***In vitro* and *in vivo* antidiabetic potential of extracts and a furostanol** **saponin from *Balanites aegyptiaca***

**Abstract**

***Context:*** *Balanites aegyptiaca* Del. (Zygophyllaceae) fruits are a well known antidiabetic drug in Egyptian folk medicine. Nevertheless, its mechanism of action is still unclear.

***Objectives***: Searching for the possible mechanisms of action of the plant and identification of the bioactive compounds.

***Materials and methods***: A bio-guided protocol based on the evaluation of α‐glucosidase (AG) and aldose reductase (AR) inhibitory activities was adopted to isolate the biologically active compounds from the methanol extract (MeEx). An *in vivo* antidiabetic study was conducted for the active extract, fraction and compound using streptozotocin-induced diabetic male albino Wistar rats at two dose levels (100 and 200 mg/kg.b/wt) for two weeks.

***Results:*** Three compounds were isolated and identified: a sterol, (**1**) stigmasterol-3-*O*-β-ᴅ-glucopyranoside; a pregnane glucoside, (**2**) pregn-5-ene-3β,16β,20(R)-trio1-3-*O*-β-ᴅ-glucopyranoside; a furostanol saponin, (**3**) 26-(*O*-β-ᴅ-glucopyranosyl)-22-*O*-methylfurost-5-ene-3β,26-diol-3-*O*-β-ᴅ-glucopyranosyl-(1→4)-[α-ᴌ-rhamnopyranosyl-(1→2)]-β-ᴅ-glucopyranoside. Only compound **3** possessed significant AG and AR inhibitory activities (IC_50_ = 3.12 ± 0.17 and 1.04 ± 0.02 µg/mL, respectively), while compounds **1** and **2** were inactive. The *in vivo* antidiabetic study revealed that MeEx and furostanol saponin **3** possessed significant activities at a dose of 200 mg/kg through reducing the fasting plasma glucose level by 46.14 and 51.39%, respectively, as well as reducing the total cholesterol by 24.44 and 31.9%, respectively. The furostanol saponin **3** also caused increment in both the insulin and C-peptide levels by 63.56 and 65%, respectively.

***Discussion and Conclusion:*** We presented a scientific base for using *Balanites aegyptiaca*, and shed the light on one of its saponins, as an antidiabetic agent in fasting and postprandial hyperglycemia along with the improvement of diabetic complications.

**Keywords:** Diabetic complications, pregnanes, insulin, C-peptides, desert date.

***Spectral data of the isolated compounds***

*Compound* ***1***

EIMS (70 eV rel. int.), *m*/*z* (%) = *m/z* at 412 [M]^+^ (100%), 399 (43%), 396 (65%), 369 (73%), 329 (17%), 271 (26%) and 255 (52%).

^1^H-NMR: δ (400 MHz, DMSO) 0.69 (3H, d, *J* = 5.4 Hz, Me-21), 0.80 (3H, t, *J* = 6.3, Me-29), 0.89 (3H, d, *J* = 6.4 Hz, Me-26), 0.92 (3H, d, *J* = 6.3 Hz, Me-27), 1.04 (3H, s, Me-18), 1.27 (3H, s, Me-19), 3.49 (1H, m, H-3), 4.21 (1H, d, *J* = 7.5, H-1 ˋ), 5.15 (dd, 1H, *J* = 8.3, 15.4, H-22), 5.21 (1H, dd, *J* = 8, 15.2, H-23) and 5.4 (1H, br.s., H-6) ppm.

*Compound* ***2***

^1^H-NMR: δ (400 MHz, DMSO) 0.91 (3H, s, Me-18), 1.01 (3H, s, Me-19), 1.21 (3H, d, *J* = 6.4 Hz, Me-21), and 5.18 (1H, d, *J* = 7.0 HZ, H-1ˋ) ppm.

The ^13^C NMR data of **1** and **2** are presented in Table 1S and the ^1^H NMR and ^13^C NMR data of **3** are presented in Table 2S.

**Table 1S.** ^13^C NMR chemical shifts (δ ppm) for compounds **1** and **2** (DMSO-d_6_, 100 MHz)

| **C** | **1** | **2** |
| --- | --- | --- |
| **1** | 38.2 | 37.2 |
| **2** | 33.3 | 30.1 |
| **3** | 77.3 | 71.3 |
| **4** | 36.6 | 37.0 |
| **5** | 140.9 | 140.9 |
| **6** | 121.6 | 121.6 |
| **7** | 31.8 | 31.5 |
| **8** | 31.8 | 31.2 |
| **9** | 50.2 | 50.8 |
| **10** | 36.5 | 36.9 |
| **11** | 21.0 | 21.2 |
| **12** | 39.6 | 38.8 |
| **13** | 42.2 | 41.5 |
| **14** | 56.8 | 54.8 |
| **15** | 24.4 | 35.2 |
| **16** | 28.9 | 73.7 |
| **17** | 55.9 | 63.0 |
| **18** | 12.0 | 15.5 |
| **19** | 19.3 | 19.0 |
| **20** | 40.5 | 67.1 |
| **21** | 21.3 | 23.7 |
| **22** | 138.3 |  |
| **23** | 129.1 |  |
| **24** | 51.1 |  |
| **25** | 31.9 |  |
| **26** | 19.0 |  |
| **27** | 21.1 |  |
| **28** | 25.4 |  |
| **29** | 12.2 |  |
| **1`** | 101.2 | 102.2 |
| **2`** | 70.3 | 70.4 |
| **3`** | 76.3 | 76.7 |
| **4`** | 73.7 | 73.1 |
| **5`** | 76.2 | 76.6 |
| **6`** | 61.8 | 61.2 |

**Table 2S:** ^1^H and ^13^C NMR chemical shifts (δ ppm) for compounds **3**, DMSO-d_6_, at 400 MHz (^1^H) and 100 MHz (^13^C)

| **Position** | **3** | |
| --- | --- | --- |
|  | **δ_C_** | **δ_H_** |
| **1-CH_2_** | 36.3 | 1.08, 1.87 (m) |
| **2-CH_2_** | 31.0 | 1.90, 1.60 (m) |
| **3- CH** | 79.2 | 3.58 (m) |
| **4- CH_2_** | 39.5 | 2.43, 2.31 (each br.s) |
| **5- C** | 141.9 | - |
| **6- CH** | 122 | 5.36 (br.s) |
| **7- CH_2_** | 33.4 | 2.00, 1.56 (m) |
| **8-CH** | 32.6 | 1.67 (dd, J=3.65 & 3.65 Hz) |
| **9- CH** | 51.5 | 0.97 (d, J=3.63) |
| **10- C** | 38.0 | - |
| **11- CH_2_** | 22.2 | 1.57,1.50 (m) |
| **12- CH_2_** | 40.1 | 1.77, 120 (m) |
| **13-C** | 41.7 | - |
| **14- CH** | 57.7 | 1.13 (m) |
| **15- CH_2_** | 33.0 | 1.97, 1.29 (m) |
| **16- CH** | 82.5 | 4.35 (br.s) |
| **17- CH** | 65.1 | 1.73 (br.s) |
| **18- CH_3_** | 16.9 | 0.82 (s) |
| **19- CH_3_** | 20.0 | 1.04 (s) |
| **20- CH** | 41.3 | 2.18 (m) |
| **21-CH_3_** | 16.0 | 1.00 (d, J=6.7 Hz) |
| **22- C** | 113.7 | - |
| **23- CH_2_** | 31.2 | 1.63, 1.80 (m) |
| **24- CH_2_** | 29.1 | 1.15, 1.59 (m) |
| **25- CH** | 34.7 | 1.74 (br.s) |
| **26- CH_2_** | 76.0 | 3.39, 3.73 (m) |
| **27- CH_3_** | 17.1 | 0.94 (d, J=6.7 Hz) |
| **OMe** | 47.5 | 3.14 (s) |
| **1`** | 100.4 | 4.51 (d, J=7.6 Hz) |
| **2`** | 78.7 |  |
| **3`** | 77.8 |  |
| **4`** | 81.0 |  |
| **5`** | 76.2 |  |
| **6`** | 62.0 |  |
| **1``** | 104.5 | 4.40 (d, J=7.6 Hz) |
| **2``** | 75.1 |  |
| **3``** | 77.8 |  |
| **4``** | 71.5 |  |
| **5``** | 77.7 |  |
| **6``** | 62.5 |  |
| **1```** | 102.0 | 5.24 (d, J=1.2 Hz) |
| **2```** | 72.3 |  |
| **3```** | 72.2 |  |
| **4```** | 74.2 |  |
| **5```** | 69.5 |  |
| **6```** | 17.9 | 1.05 (d, J=6.7 Hz) |
| **1````** | 104.4 | 4.24 (d, J=7.7 Hz) |
| **2````** | 75.0 |  |
| **3````** | 71.3 |  |
| **4````** | 78.0 |  |
| **5````** | 78.0 |  |
| **6````** | 62.3 |  |
